# Drilling Characteristics of Additively Manufactured PLA/17-4 PH Stainless Steel Hybrid Composite: Thrust Force, Surface Roughness, Vibration and Temperature Change

**DOI:** 10.3390/polym18121434

**Published:** 2026-06-08

**Authors:** Erhan Şentürk, Cem Alparslan, Ramazan Ötüken, Muhammed Furkan Erhan, Şenol Bayraktar

**Affiliations:** 1 Department of Mechanical Engineering, Faculty of Engineering and Architecture, Recep Tayyip Erdoğan University, Rize 53100, Türkiye; erhan_senturk20@erdogan.edu.tr (E.Ş.); cem.alparslan@erdogan.edu.tr (C.A.); senol.bayraktar@erdogan.edu.tr (Ş.B.); 2 FDM and Metallography Laboratory, Department of Mechanical Engineering, Recep Tayyip Erdoğan University, Rize 53100, Türkiye; 3 Department of Electronic Automation, Vocational School of Technical Sciences, Recep Tayyip Erdoğan University, Rize 53100, Türkiye; 4 Department of Manufacturing Engineering, Faculty of Technology, Gazi University, Ankara 06560, Türkiye; furkanerhan@gazi.edu.tr

**Keywords:** hybrid additive manufacturing, drilling, vibration, composite material, FDM

## Abstract

Understanding the finishing behavior of hybrid structures produced by additive manufacturing based on FDM is critically important in systems where phases with different thermal and mechanical properties coexist. In this study, the drilling performance of hybrid structures with a PLA/17-4 PH/PLA layer arrangement was comprehensively investigated in terms of thrust force, moment, surface roughness, temperature variation, vibration behavior, and surface integrity. For this purpose, a total of 16 drilling tests were performed on 56 × 56 × 15 mm hybrid specimens with 100% infill density, in a full factorial configuration, at cutting speeds (*V*) of 80–170 m/min and feed rates (*f*) of 0.04–0.16 mm/rev. The middle layer was used in the as-printed green state as a 17-4 PH metal-filled filament containing metal particles and binder, without any debinding or sintering step. The results showed that increasing feed rate increased thrust force, moment, and surface roughness in all layers, whereas increasing cutting speed decreased these values and promoted a more stable drilling regime. The middle 17-4 PH layer exhibited lower surface roughness than the outer PLA layers, while thermal measurements indicated limited variation at the hole entrance and higher temperature accumulation at the hole exit. The most favorable drilling condition within the studied hybrid configuration was obtained at 170 m/min and 0.04 mm/rev, whereas the least favorable condition was obtained at 80 m/min and 0.16 mm/rev. Overall, the combination of high cutting speed and low feed rate provided the most suitable drilling window for the studied hybrid structure. The findings also indicated that surface quality was more strongly associated with cutting load and high-frequency vibration components than with vibration level alone.

## 1. Introduction

Conventional manufacturing technologies have long played a fundamental role in engineering production. However, they present several limitations in the fabrication of complex geometries, the design of lightweight structural components, and the manufacturing of multi-material systems. These limitations restrict design flexibility and impose increased cost and time constraints on manufacturing processes. In recent years, rapidly advancing additive manufacturing (AM) has offered a paradigm shift in production by enabling the layer-by-layer fabrication of complex geometries, thereby overcoming the limitations of conventional subtractive manufacturing methods [1,2]. One of the most widely used methods of AM is Fused Filament Fabrication (FFF), also known as Fused Deposition Modeling (FDM). This method is based on the controlled melting and layer-by-layer deposition of thermoplastic filament. As a result, it offers advantages such as low cost, equipment accessibility, process flexibility, and reduced material waste during production [3,4,5]. Owing to its user-friendly nature, wide material range (PLA—polylactic acid, PETG—polyethylene terephthalate glycol, ABS—acrylonitrile butadiene styrene, ASA—acrylonitrile styrene acrylate, PA—polyamide, PC—polycarbonate, TPE—thermoplastic elastomer, PVA—polyvinyl alcohol, and metal-filled filaments), and low initial cost, FDM technology has been widely adopted in applications ranging from industrial production to academic research [6,7,8,9]. It is widely used in industrial, biomedical, and functional prototyping applications due to its versatility, accessibility, and material efficiency [2,10,11,12,13,14]. In academic studies, FDM materials are commonly evaluated in terms of mechanical, thermal, structural, and machinability-related properties [15,16,17,18]. Within this broad material ecosystem and academic research landscape, thermoplastic polymers have gained prominence. Among thermoplastic polymers, polylactic acid (PLA) is one of the most widely used FDM materials due to its print stability, ease of processing, bio-based origin, and biodegradable nature, which support its broad use in both prototyping and engineering applications [19,20,21,22,23,24,25,26]. However, in its pure form, PLA may be mechanically insufficient for applications requiring high strength [27,28,29]. This limitation is being addressed through hybrid manufacturing strategies involving the combined use of different materials and reinforced filament technologies. Among these approaches, the development of metal-filled filaments is of particular interest, as it allows FDM systems to go beyond polymer-based prototyping and enables the production of metallic or metal-reinforced components. In recent years, the development of metal-filled filaments has significantly broadened the application range of this field by allowing conventional FDM systems to be adapted to produce metallic parts [30,31]. BASF Ultrafuse 17-4 PH is a metal-filled filament based on 17-4 PH precipitation-hardening stainless steel and represents an important route for extending FDM toward metal-containing components. Its processing concept is similar to Metal Injection Molding (MIM); however, in the present study, the material was used only in the green state, without debinding or sintering, and therefore should not be interpreted as a fully metallic core [32,33,34]. Therefore, the middle layer should be interpreted as a green-state metal-filled filament region rather than as a dense metallic phase. The green-state configuration was intentionally selected in this study because many FDM-produced metal-filled structures may require secondary machining operations before debinding or sintering, particularly during prototype development, dimensional correction, assembly preparation, or hybrid multi-material manufacturing stages. In addition, the drilling response of green-state structures differs fundamentally from that of fully sintered metallic parts due to their binder-rich composition, heterogeneous interfacial structure, reduced stiffness, and anisotropic layer-dependent behavior. These characteristics create a distinct machining environment involving unstable chip formation, localized deformation, and complex thermal–mechanical interactions. Despite its practical relevance, the drilling behavior of green-state hybrid architectures remains insufficiently investigated in the literature. Therefore, the present study specifically focuses on clarifying the drilling response of an as-printed heterogeneous layered structure rather than evaluating the behavior of a fully densified metallic system.

The combination of different material classes such as PLA and 17-4 PH in the form of FDM-based hybrid structures brings hybrid manufacturing approaches to the forefront. In our previous study on the same material system, this layered concept was introduced primarily to explore the architectural and interfacial behavior of a green-state hybrid configuration rather than to surpass monolithic PLA in tensile performance. That study showed that pure PLA exhibited the highest tensile properties, whereas the hybrid structures displayed intermediate behavior depending on layer arrangement and infill architecture [35]. This approach enables the design of functionally differentiated layered structures with distinct interfacial and machining behavior. Depending on the material configuration, such systems may offer advantages in terms of weight reduction and functional design flexibility [36,37,38]. Beyond serving as a drilling behavior research platform, PLA/17-4 PH/PLA sandwich architectures may also have potential relevance for functional prototyping applications. Although the green-state 17-4 PH-containing layer should not be regarded as a fully metallic or mechanically superior core, the presence of metal particles within the intermediate layer may provide functional benefits such as localized mass increase, stiffness tailoring, magnetic response, electromagnetic interference shielding, thermal moderation, or functionally graded material behavior. Such polymer/metal-filled/polymer configurations may therefore be useful in prototype components where functional differentiation between layers is desired. However, these potential application scenarios were not experimentally evaluated in the present study and should be considered as future research directions rather than confirmed performance outcomes. In the present study, this hybrid architecture was selected not to claim superiority over pure PLA, but to create a heterogeneous drilling path for investigating interfacial machining behavior, cutting-load fluctuations, temperature accumulation, and vibration response in a green-state multi-material FDM structure. However, the potential functional benefits of hybrid structures are not sufficient on their own because surface roughness, dimensional accuracy, and geometric tolerance limitations inherent to the AM process still require attention. This necessitates the application of post-processing operations such as drilling in conventional machining to achieve critical features requiring high precision, such as assembly holes [39,40]. During conventional drilling operations applied to different materials, issues such as high cutting forces, elevated temperature generation, and uncontrolled deformation may occur. This situation leads to more complex machining behavior, particularly in hybrid structures where materials such as PLA and 17-4 PH, which have different thermal and mechanical properties, are combined. The mechanical and thermal incompatibility between these materials results in irregular cutting mechanics, accelerated tool wear, and deterioration of surface integrity, thereby making drilling performance unpredictable [41,42,43,44]. In this context, drilling parameters play a critical role in minimizing mechanical and thermal mismatches, optimizing cutting mechanics, limiting tool wear, and preserving surface integrity. The selection of appropriate drilling parameters results in a substantial reduction in cutting forces, improved chip formation and fracture behavior, and enhanced surface quality. These improvements contribute to controlling thermal and mechanical effects in hybrid structures such as PLA/17-4 PH, thereby enabling the preservation of surface integrity and providing an effective post-processing approach for achieving more reliable hole quality.

In the literature, several studies have examined the drilling behavior of PLA-based and reinforced FDM structures under different machining and material conditions. Sneha and Balamurugan [45] investigated micro-drilling of PLA–bronze composite filament and showed that process parameters strongly affected delamination behavior. Vishwadarshan et al. [46] evaluated drilled PLA parts produced under different printing and machining conditions and identified parameter combinations that minimized delamination. Shunmugesh et al. [47] reported that spindle speed, feed rate, and drill diameter significantly influenced surface roughness and hole quality in PLA filament parts. Eşiyok and Ergene [48] showed that thrust force increased with feed rate and drill diameter, while higher cutting speed reduced force levels in PLA and PLA/CF structures. Kumar and Jayakumar [49] found that spindle speed and feed rate were the dominant factors affecting delamination and roughness in reinforced PLA composites, whereas Masannan et al. [50] reported that feed rate was the most influential parameter on thrust force in kenaf fiber-reinforced PLA. Overall, the reviewed studies show that drilling performance in PLA-based and reinforced FDM structures is mainly governed by cutting speed, feed rate, drill geometry, and material composition. In general, increasing feed rate leads to higher cutting forces, roughness, and damage formation, whereas higher cutting speed tends to improve hole quality under suitable conditions. However, most previous studies focused on monolithic PLA or PLA-based composites, and the drilling behavior of layered green-state hybrid architectures involving polymer/metal-filled transitions remains insufficiently addressed.

Nevertheless, the available literature on the drilling performance of FDM-fabricated PLA/17-4 PH hybrid structures is still limited, particularly for green-state layered architectures at material transition zones. This issue remains relevant because multi-material components may still require secondary machining for assembly and functional integration. Accordingly, there is still a lack of studies that holistically assess drilling performance in such multi-phase and layered structures by considering interrelated parameters such as force, torque, temperature, vibration, and surface quality, rather than focusing solely on individual outputs. However, in hybrid composite structures produced by FDM, factors such as anisotropy induced by layer-by-layer fabrication, variations in interlayer adhesion, internal void formation, the distribution of metal particles within the polymer matrix, and mechanical incompatibilities at the metal–polymer interface can play a decisive role in cutting resistance, energy consumption, thermal load accumulation, dynamic stability, and surface integrity during drilling. Therefore, a detailed investigation of the machinability behavior of such hybrid systems constitutes an important research need, both for understanding post-production secondary machining processes and for determining optimal processing conditions for practical applications. This study aims to comprehensively evaluate the drilling behavior of PLA/17-4 PH hybrid composite structures produced by FDM. It is intentionally limited to the as-printed green-state hybrid configuration and does not seek to demonstrate superiority over monolithic PLA; instead, it focuses on identifying the drilling response and the most suitable machining window within this specific layered architecture. In this context, within the scope of this study, the effects of the interlayer bonding characteristics, anisotropic mechanical response, and metal–polymer phase interactions of the hybrid structure on drilling performance will be investigated through key performance outputs such as thrust force, torque, process temperature, vibration level, and surface roughness. Furthermore, the linear, nonlinear, and interaction effects of drilling parameters, especially *V* and *f*, on the output variables are intended to be investigated. In addition to conventional single-response analyses, Principal Component Analysis (PCA) is employed as a multivariate statistical approach to reveal the underlying relationships among the drilling performance indicators, reduce the dimensionality of the dataset, and identify the dominant factors governing the overall machining behavior of the hybrid structure.

## 2. Materials and Methods

**Figure 1:**
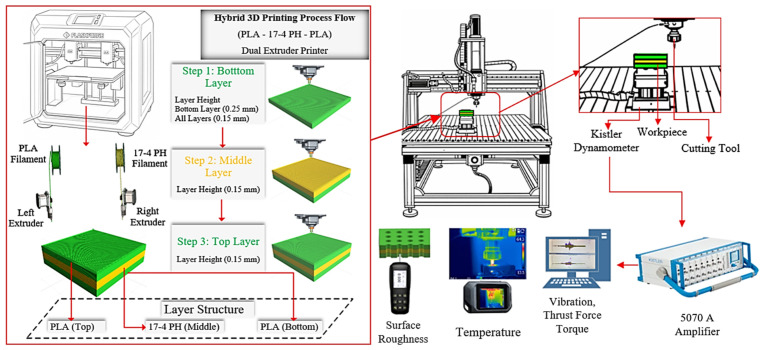
Overall experimental workflow, including hybrid specimen fabrication, drilling experiments, and measurement and analysis stages.

This study is based on an experimental methodology aimed at investigating the drilling behavior of hybrid PLA/17-4 PH/PLA structures produced by the FDM-based AM method. The experimental procedure consists of filament preparation, fabrication of specimens with a hybrid layer architecture using a dual-extruder system, preparation of the drilling setup, and the measurement and analysis of feed force, temperature variation, vibration, and surface roughness during drilling. The overall experimental workflow, including specimen fabrication, drilling tests, and measurement/analysis stages, is schematically presented in Figure 1.

### 2.1. Materials and FDM Printing Parameters

In this study, PLA (polylactic acid) and 17-4 PH stainless steel-based Ultrafuse^®^ filaments (Ludwigshafen, Germany) were used as the thermoplastic matrix and metal-filled material, respectively, in the production of hybrid specimens (Table 1). PLA filaments were supplied by eSUN (Shenzhen, China) [51], while the 17-4 PH filament was obtained from BASF Forward AM [52]. Both filaments have a nominal diameter of 1.75 mm. PLA was preferred as the matrix phase due to its widespread use in FDM processes and its high print stability and dimensional stability. The 17-4 PH-based filament has a composite structure containing metal powder and a binder system and constitutes the metal-filled middle layer of the hybrid structure. It should be emphasized that no debinding or sintering treatment was applied after printing. Therefore, the middle layer remained in the green state throughout the experiments and should not be interpreted as a fully metallic 17-4 PH core.

The production of hybrid specimens was carried out using a Flashforge Creator 3 desktop FDM printer (Jinhua, China) with an Independent Dual Extruder (IDEX) system [53]. This system enabled the controlled formation of the hybrid layer structure by allowing PLA and 17-4 PH stainless steel-based filaments to be processed through separate extruders. The printer used in this study has technical specifications suitable for meeting the thermal and mechanical requirements of the materials used. The system is configured with extruder units operating up to 300 °C, a closed chamber structure, and a heated build platform with a capacity of 120 °C. Due to the abrasive nature of the 17-4 PH filament, a hardened steel nozzle with a diameter of 0.4 mm was used instead of a standard brass nozzle during the printing process. This selection is crucial for reducing nozzle wear and preserving geometric stability during the printing process. The printing processes were carried out under enclosed chamber and heated build plate conditions. In this way, a controlled printing environment was provided in the hybrid manufacturing process, where materials with different thermal behaviors are processed together, aiming to reduce cooling rate-induced thermal stresses and interlayer separation risks. The hybrid specimens were fabricated with a PLA/17-4 PH/PLA layer arrangement, and the selected hexagonal infill geometry was stably formed owing to the system’s precise motion control.

### 2.2. Experimental Design and Hybrid Sample Manufacture

**Figure 2:**
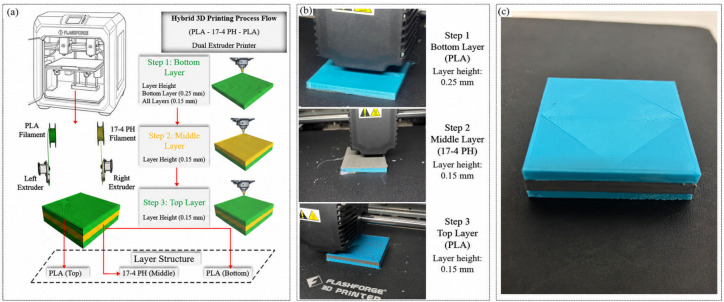
Hybrid PLA/17-4 PH/PLA fabrication process and representative printed specimens: (**a**) schematic illustration of the dual-extruder printing route and layer architecture; (**b**) sequential printing stages of the bottom PLA layer, middle 17-4 PH-containing layer, and top PLA layer during fabrication; and (**c**) final printed hybrid specimen showing the interlayer continuity and overall print quality prior to drilling experiments.

In this study, hybrid specimens with a sandwich structure were designed and fabricated based on experimental findings and optimization data from reference studies [35]. The hybrid structure was designed as a three-layer system consisting of PLA in the bottom layer, 17-4 PH stainless steel-based filament in the middle layer, and PLA in the top layer. The specimens were fabricated with dimensions of 56 × 56 × 15 mm, and each layer was designed with a thickness of 5 mm. Prior to the fabrication of the final specimens, preliminary trials were conducted to determine the appropriate printing parameters, ensuring proper interlayer bonding and structural integrity. These parameters were established based on previous work and repeated trial productions. Following this optimization stage, a total of two hybrid specimens were fabricated, and all drilling experiments corresponding to the full factorial design were performed on these specimens at different drilling locations. Each drilling parameter combination was applied once within the full factorial experimental design. Separate drilling locations were used for each condition in order to avoid the influence of previous machining operations on subsequent measurements. The experimental strategy was designed to comparatively evaluate the drilling response trends of the selected hybrid configuration under controlled machining conditions. Thus, a multilayer hybrid structure with a PLA/17-4 PH/PLA configuration was obtained, with a total part height of 15 mm. The purpose of this configuration was to establish a heterogeneous drilling path with distinct material transitions and to investigate machining behavior at polymer/metal-filled interfaces, rather than to propose a mechanically superior alternative to monolithic PLA. The production parameters were determined to maintain the interlayer bonding quality and geometric stability of the hybrid structure. In this context, a nozzle with a diameter of 0.4 mm was used for all specimens. The extrusion temperature was set to 250 °C for PLA and 270 °C for 17-4 PH. The build plate temperature was defined as 60 °C for the bottom PLA layer, 90 °C for the middle 17-4 PH layer, and 60 °C for the top PLA layer. The initial layer height was selected as 0.25 mm, whereas the remaining layers were set to 0.15 mm. The printing speed was kept constant at 65 mm/s throughout the entire production process. The specimens were fabricated with a 100% infill ratio and a hexagonal infill pattern. The hexagonal infill pattern was selected because it provides a continuous and repetitive internal cell geometry that supports structural stability and promotes a more uniform load distribution within the printed part. In the present study, this pattern was used to obtain a consistent internal architecture during drilling and to reduce geometry-related variability among the hybrid specimens. The literature also reports that a 100% dense hexagonal infill pattern is preferred for this hybrid structure, as it provides high structural integrity and mechanical strength [35]. During the printing process, raft, pre-extrusion, and wall generation features were disabled, and the cooling fan was kept off throughout the entire printing duration. Additionally, the inter-extruder calibration parameter (Z-offset) was carefully adjusted to enhance bonding quality at the interlayer transition zones. The slicing of the specimens was performed using FlashPrint 5.6.0 software, and the production process was completed using a desktop FDM-based 3D printer. Figure 2 specifically illustrates the hybrid specimen fabrication route, including the layer arrangement and printing strategy used to produce the PLA/17-4 PH/PLA structure.

### 2.3. Drilling Experiments

Drilling tests were performed on hybrid specimens with dimensions of 56 × 56 × 15 mm, produced using an FDM Flashforge Creator 3 printer, on a CNC router machine specially manufactured by Da Vinci Engineering (Ankara, Türkiye) in accordance with the requested specifications. During the drilling tests, the specimens were rigidly fixed on the machine table through the dynamometer setup in order to prevent displacement and vibration-related positioning errors during machining. The machine, operated using Mach3 3.043.066 CNC control software, has dimensions of 750 × 950 × 200 mm. The machine, constructed from aluminum profiles, has a maximum motor speed limit of 18,000 rpm. In the drilling operations, an uncoated solid carbide drill compliant with ISO 235 [54] (drill diameter: 6 mm, point angle: 118°, helix angle: 30°, cutting length: 25 mm, and total length: 50 mm) was used.

To simultaneously monitor process responses during drilling operations, a measurement system consisting of a thermal camera, dynamometer, and three-axis accelerometer was employed. In the experimental setup, the dynamometer was positioned beneath the specimen to measure thrust force and torque during drilling, the thermal camera was placed in front of the cutting zone to monitor the tool–workpiece interaction region, and the triaxial accelerometer was attached to the experimental system to record vibration response during machining. A high-resolution infrared thermal camera system (FLIR HM1100, Wilsonville, OR, USA) was used to monitor the thermal loads generated in the tool–material interaction zone. This system enabled high-accuracy recording of instantaneous temperature variations during drilling, with a resolution of 640 × 512 pixels, a recording rate above 60 Hz, and a measurement accuracy of ±2 °C. To enhance measurement accuracy, the emissivity value in the thermal camera system was defined as 0.95, and the system was calibrated under appropriate measurement conditions before the experiments. In addition, the thermal measurements were performed under constant ambient laboratory conditions and with a fixed camera position in order to minimize positional and environmental variability between experiments. The measurement uncertainty of the thermal camera was considered within the manufacturer-specified accuracy limits, and all thermal images were evaluated using the same emissivity setting and analysis procedure. After the preliminary drilling tests, the thermal camera was positioned in front of the drilling zone on the CNC machine to directly monitor the interaction region between the cutting tool and the workpiece. Temperature distributions before, during, and after each drilling operation were recorded, and the acquired images were analyzed using appropriate analysis software. Within this scope, Tmax, Tavg, and temperature distribution profiles were determined for each experiment. For consistency, the temperature data were extracted from the same analysis region for all experiments, and the reported values were compared on the basis of identical image-processing settings. During the drilling process, thrust force and torque values were measured with high precision using a Kistler 9257B dynamometer (Winterthur, Switzerland) (force range: ±5 kN, accuracy: ±0.5%). Before the experiments, the dynamometer system was checked and zero-balanced according to the manufacturer’s recommendations to ensure reliable force acquisition. Possible measurement deviations were considered within the specified system accuracy, and the same acquisition setup was maintained throughout all drilling tests. The signals obtained during measurement were processed via a Kistler 5070A charge amplifier module (torque resolution: 0.01 N·m), and signal integrity was maintained throughout the data acquisition process. The recorded force and torque signals were evaluated using the same data acquisition conditions for all experiments, and the characteristic response levels were determined from the processed drilling signals obtained during tool–workpiece interaction. In addition, a Dytran 3273A2 triaxial accelerometer (Chatsworth, CA, USA) was used in experiments to evaluate vibration behavior. The accelerometer was mounted at a fixed location in the experimental setup to ensure consistent acquisition of vibration signals throughout all drilling tests. Prior to the tests, the sensor installation and signal response were checked to ensure stable measurement conditions. All vibration measurements were collected using the same sensor position and acquisition setup in order to maintain comparability among the experiments. Through this system, acceleration data was obtained in the time domain, and subsequently, Fast Fourier Transform (FFT) was applied to reveal the frequency components of the vibration signals. The vibration signals were processed under identical analysis conditions for all experiments. Time-domain data were first used to determine overall vibration levels, and then frequency-domain analysis was performed through FFT to evaluate the distribution of vibration energy in different frequency bands.

**Figure 3:**
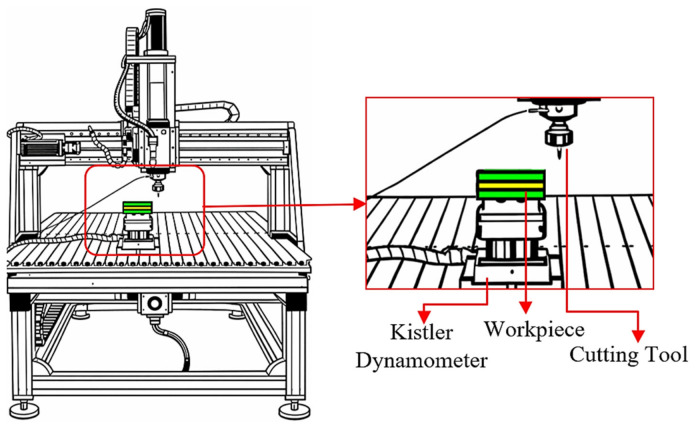
Drilling test setup.

In the experimental design, *V* and *f* were selected as independent variables. A full factorial experimental design was applied for these two parameters, and a total of 16 drilling experiments were conducted. Drilling experiments were conducted under different *Vs* (80, 110, 140, and 170 m/min), *fs* (0.04, 0.08, 0.12, and 0.16 mm/rev), and a constant drilling depth of 15 mm. The cutting parameters used in the study were determined based on both the appropriate ranges reported in the literature and observations obtained from preliminary experiments [46,48,55]. In addition, preliminary experiments were conducted to verify tool stability and to confirm that excessive vibration, chip accumulation, or tool breakage did not occur under the selected operating conditions. Based on the obtained findings, the selected parameter combinations were considered suitable for reliable data acquisition and comparison with previous studies. The drilling experimental setup is presented in Figure 3.

After the drilling process, to determine the surface roughness characteristics of the machined hole surfaces, the specimens were sectioned along the hole axis using a precision cutting method. With this procedure, the inner hole surfaces were made accessible for measurement, and the two opposite cross-sectional surfaces of each specimen were evaluated separately. Surface roughness measurements were performed according to ISO 4287 [56] using a MAHR Perthometer stylus-based surface roughness device (Göttingen, Germany). For each hole surface, measurements were taken at three different points, and the data obtained from both surfaces were recorded separately, with the arithmetic mean of these measurements used as the average surface roughness (Ra) value. By selecting measurement points from different regions, it was aimed to characterize local variations in surface roughness within the inner hole surface. In all measurements, identical experimental and data-processing procedures were followed in order to reduce systematic error and ensure comparability among the drilling conditions. The reported results were interpreted within the accuracy limits of the respective instruments. Within the scope of this study, the main measured and evaluated quantities were thrust force (Fz), torque (Mz), surface roughness (Ra), maximum and average temperature values, and vibration response parameters obtained from time- and frequency-domain analyses. These variables were used as the main indicators of drilling performance for the hybrid structure.

### 2.4. Hole Inner Surface Quality

A Dino-Lite AM4113ZT digital microscope (New Taipei City, Taiwan) was employed after drilling to examine the machined surface, edge damage around the holes, chip morphology, and the condition of the drill cutting edges. Following drilling, the inner surfaces of the holes, the entry and exit regions, and the cross-sectional surfaces of the specimens were examined in detail. The evaluations considered burr formation, plastic deformation marks, interlayer delamination, void and porosity structures, filament traces, micro-crack initiation sites, and crack propagation paths. In addition, the chips generated during drilling were collected and evaluated in terms of their shape, fracture characteristics, and overall structural features. The obtained imaging findings enabled a qualitative comparison of the effects of drilling parameters on machined surface quality and surface integrity. Furthermore, the machinability behavior of the hybrid layered structure was assessed by correlating the damage mechanisms at the hole entry and exit regions with the measured thrust force, torque, and vibration characteristics. After drilling, edge damage, matrix cracking, interlayer separation tendencies, surface irregularities, and chip formation characteristics were evaluated in correlation with the measured mechanical responses.

### 2.5. Principal Component Analysis (PCA)

In order to identify the relationships between the drilling performance parameters and to reduce the dimensionality of the dataset, Principal Component Analysis (PCA) was performed. The analysis was conducted using thrust force (Fz), torque (Mz), and surface roughness (Ra) values obtained from the top, middle, and bottom layers, together with maximum temperature (Tmax) and high-frequency vibration component (E_high). Prior to the analysis, all variables were standardized, and the PCA was carried out based on the correlation matrix to eliminate the effect of different measurement units. The analysis was performed using Minitab 19.0 statistical software. The number of principal components to be retained was determined according to the Kaiser criterion (eigenvalue > 1). In addition, scree plot analysis was used to support the selection of significant components. PCA results were interpreted through loading and score plots to evaluate variable relationships and clustering behavior of experimental conditions.

## 3. Results and Discussion

### 3.1. Drilling Performance Indicators: Thrust Force, Torque, and Surface Roughness

**Figure 4:**
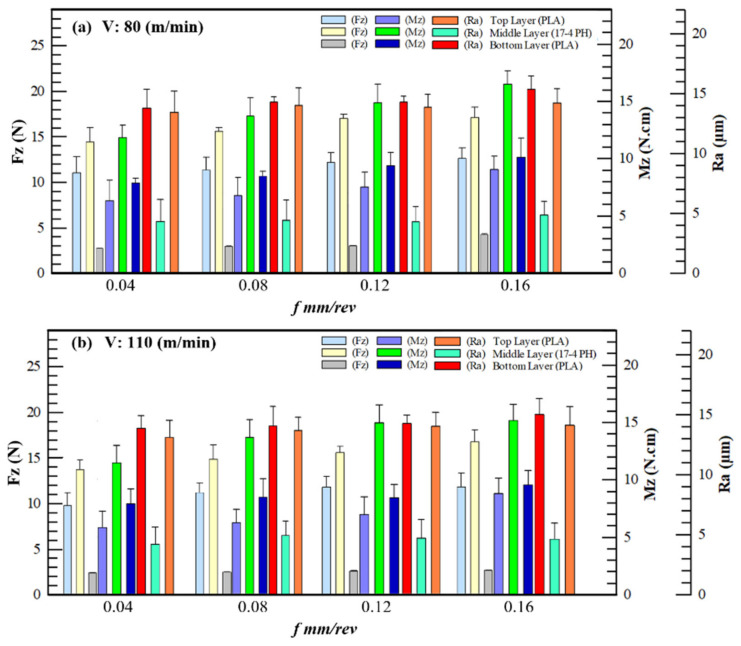

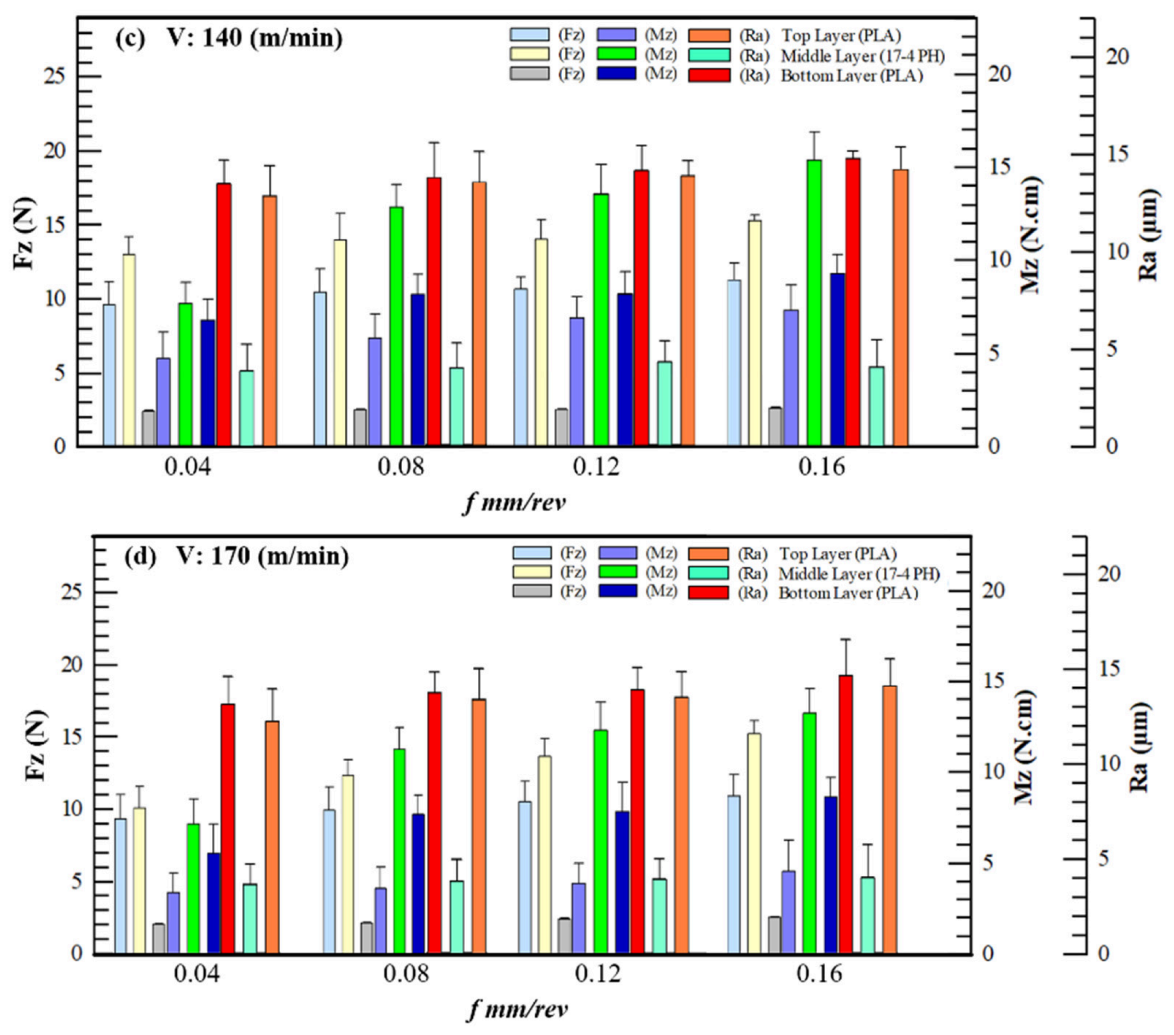
Changes in the drilling performance indicators of the hybrid PLA/17-4 PH/PLA structure under different *V* and *f* combinations: thrust force (Fz), torque (Mz), and surface roughness (Ra) at *V*s of (**a**) 80 m/min, (**b**) 110 m/min, (**c**) 140 m/min, and (**d**) 170 m/min.

The thrust force (Fz), moment (Mz), surface roughness (Ra), vibration, and temperature values generated during the drilling process can in general be affected by many factors reported in the literature, such as material structure, infill characteristics, layer height, tool geometry, spindle speed, and feed rate. In the present study, however, the experimental evaluation was specifically focused on the effects of cutting speed (V) and feed rate (f) within the selected hybrid specimen configuration. These variables play a critical role in determining the mechanical and thermal response of the PLA/17-4 PH hybrid structure produced by the FDM method during drilling. The literature also reports that the drilling outputs in PLA-based structures produced by AM are sensitive to the combined effect of production and processing parameters [46]. Figure 4 shows the time-dependent variation in thrust force and moment values during the drilling process. During drilling, the drill bit contacts the upper PLA layer, the middle 17-4 PH layer, and the lower PLA layer in sequence; Fz and Mz values increase with material entry, and these values approach zero again when the tool completely exits the material. The fact that the force and moment values, which are initially zero, increase with the onset of tool-material interaction and then decrease again at the exit clearly demonstrates the drilling behavior due to the layered architecture of the hybrid structure. Before discussing the results, it should be noted that the present findings describe parameter-dependent trends only within the selected green-state hybrid architecture. Since no monolithic PLA drilling control was included in the experimental design, the results should not be interpreted as evidence that the hybrid configuration provides superior drillability or surface quality relative to pure PLA.

**Figure 5:**
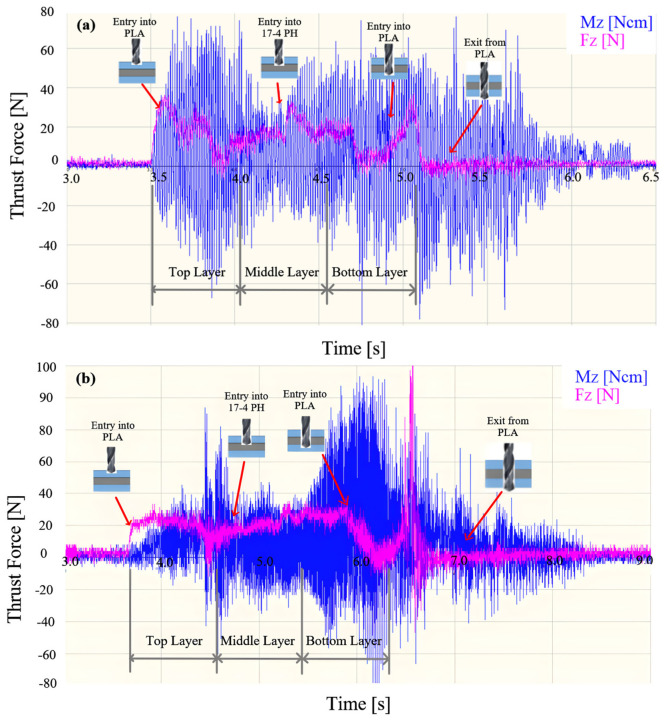
Time-dependent variation in thrust force (Fz) and torque (Mz) during drilling of the hybrid PLA/17−4 PH/PLA structure: (**a**) V = 80 m/min, f = 0.16 mm/rev, and (**b**) V = 170 m/min, f = 0.04 mm/rev.

It was determined that increasing the *f* from 0.04 mm/rev to 0.16 mm/rev at constant *V*s (80, 110, 140 and 170 m/min) increased the Fz, Mz and Ra values in all layers (Figure 4a–d). Figure 4 clearly shows that the Fz values systematically increase in all layers with increasing *f*. For consistency, the layer sequence in Figure 4 corresponds to the top PLA layer, middle 17-4 PH layer, and bottom PLA layer. Although surface roughness (Ra) values are presented on a secondary axis, the same layer order is preserved and should be interpreted accordingly. This trend can be explained by the fact that the increase in *f* increases the volume of chips removed by the cutting tool in one revolution, and consequently, the chip cross-sectional area increases. This increase in chip cross-sectional area increases the resistance of the material to deformation; consequently, the energy requirement per unit time increases during the drilling of the hybrid structure, and higher cutting forces are generated. The literature also reports that increasing the *f* significantly increases the thrust force in PLA-based and biocomposite structures produced with FDM [48,50,57]. In contrast, a general tendency to decrease was observed in all response variables with increasing *V*. This can be attributed to the cutting mechanism becoming relatively more stable at higher *V*s, the reduction in tool-workpiece contact time, and the more regular material removal process. As a result of evaluating the Fz, Mz, and Ra values together, it was determined that the most favorable drilling condition within the studied hybrid configuration was obtained in test condition 13 (170 m/min, 0.04 mm/rev), while the least favorable condition was obtained in test condition 4 (80 m/min, 0.16 mm/rev). This contrasting behavior is also clearly seen in the time-dependent force and moment signals, with a lower and more stable loading character observed in test 13, while a higher and more fluctuating signal structure emerged in test 4 (Figure 5). The prominent peak observed in the Fz signal corresponds to the transition of the drill into the higher-resistance middle 17-4 PH-containing layer, where the cutting load temporarily increases due to the change in material response and interfacial interaction.

At a *V* of 80 m/min (Figure 4a), Fz values were measured in the range of 10.97 to 12.74 N in the top PLA layer, 14.35 to 17.28 N in the middle 17-4 PH layer, and 2.78 to 4.21 N in the bottom PLA layer. Under the same conditions, Mz values ranged from 6.66 to 9.07 N·cm in the top PLA layer, 12.40 to 16.53 N·cm in the middle 17-4 PH layer, and 8.28 to 10.16 N·cm in the bottom PLA layer. Ra values were determined in the range of 13.872 to 15.4465 µm in the top PLA layer, 4.35575 to 4.88675 µm in the middle 17-4 PH layer, and 13.5382 to 14.2962 µm in the bottom PLA layer. At a *V* of 110 m/min (Figure 4b), Fz values were measured as 9.78 to 11.99 N in the top PLA layer, 13.69 to 16.98 N in the middle 17-4 PH layer, and 2.47 to 2.78 N in the bottom PLA layer, respectively. At the same rate, Mz values were calculated to be in the range of 5.83 to 8.84 N·cm in the top PLA layer, 11.39 to 15.18 N·cm in the middle layer, and 7.88 to 9.59 N·cm in the bottom PLA layer. Ra values were obtained as 13.7895 to 14.984 µm in the top PLA layer, 4.254 to 4.687 µm in the middle layer, and 13.046 to 14.111 µm in the bottom PLA layer.

At a *V* of 140 m/min (Figure 4c), the Fz values for the top PLA, middle 17-4 PH, and bottom PLA layers ranged from 9.55 to 11.19 N, 12.92 to 15.20 N, and 2.39 to 2.58 N, respectively. Under the same conditions, the Mz values were determined as 4.59 to 7.35 N·cm for the top PLA layer, 7.44 to 15.44 N·cm for the middle layer, and 6.59 to 9.31 N·cm for the bottom PLA layer. The Ra values were measured in the range of 13.316 to 14.6258 µm for the top PLA layer, 3.8452 to 4.063 µm for the middle 17-4 PH layer, and 12.7197 to 14.052 µm for the bottom PLA layer. At a *V* of 170 m/min (Figure 4d), Fz values were found to range from 9.32 to 10.87 N in the top PLA layer, 10.05 to 15.11 N in the middle 17-4 PH layer, and 2.13 to 2.57 N in the bottom PLA layer. Similarly, Mz values were calculated to range from 3.44 to 4.59 N·cm in the top PLA layer, 7.19 to 13.23 N·cm in the middle layer, and 5.58 to 8.64 N·cm in the bottom PLA layer. At the same rate, Ra values were measured as 12.8754 to 14.571 µm in the top PLA layer, 3.635 to 4.00 µm in the middle 17-4 PH layer, and 11.993 to 14.00 µm in the bottom PLA layer.

It has been observed that the increase in *f* creates a significant boosting effect not only on Fz but also on Mz. The increase in the chip cross-sectional area increases the cutting resistance, leading to an increase in the drilling moment. Furthermore, due to the multilayered and heterogeneous character of the hybrid structure, the activation of different deformation mechanisms in different layers during drilling increases the tendency for friction and adhesion at the tool-workpiece interface, contributing to an increase in rotational resistance. This trend is consistent with previous studies investigating the drilling behavior in composite and biodegradable-based structures [49,58]. When comparing the layers, the fact that the highest Fz and Mz values are mostly obtained in the middle 17-4 PH layer clearly shows that this region has higher cutting resistance. In contrast, the lower force levels determined in the lower PLA layer can be explained by the decrease in material support as the drill approaches the exit zone and the change in effective contact conditions in the cutting zone.

Surface roughness results also increased in parallel with the increase in *f*. Increased *f* negatively affects hole surface quality by causing larger chip formation and more irregular deformations during cutting. As a result, tearing, microcrack formation, and irregular topography can develop on hole surfaces. Furthermore, the increase in vibration amplitude in the system depending on the *f* is also an important factor that increases surface roughness [59,60,61,62]. In the literature, the relationship between surface roughness and *f* is ideally expressed by the equation:

(1) Ra= f232r 

(where *f* is feed rate and r is tool nose radius), which shows that the increase in *f* increases surface roughness quadratically. It can be said that the obtained experimental findings are consistent with this theoretical expectation [63]. However, the fact that Ra values are higher in the upper and lower PLA layers and lower in the middle 17-4 PH layer shows that the surface formation mechanisms depending on the layers differ from each other. The fact that effects such as plastic deformation, material scaling, and surface tearing become more dominant in polymer-based regions may have led to higher roughness values in these layers. When the obtained results are compared with previous studies on PLA and PLA-based composites, it is observed that the increasing trend of thrust force and surface roughness with feed rate and the decreasing trend with cutting speed are consistent with the general behavior reported in the literature [48,49,50,57]. However, the layered hybrid architecture examined in this study introduces additional complexity due to material transitions, which leads to more pronounced variations in cutting response compared to single-material systems. This indicates that interfacial effects and heterogeneous material behavior play a critical role in machining performance.

In order to better interpret the relationship between drilling loads and surface quality, a qualitative trend-based assessment was performed among thrust force (Fz), torque (Mz), and surface roughness (Ra). The results suggest that higher Fz and Mz values generally coincided with increased Ra values, indicating that increased cutting loads may contribute to surface deterioration. However, since no additional Pearson or Spearman correlation analysis was performed, this relationship was interpreted as a qualitative tendency rather than a statistically confirmed strong correlation. Therefore, this relationship should be interpreted cautiously as a qualitative tendency rather than as a statistically confirmed correlation.

### 3.2. Thermal Behavior During Drilling

**Figure 6:**
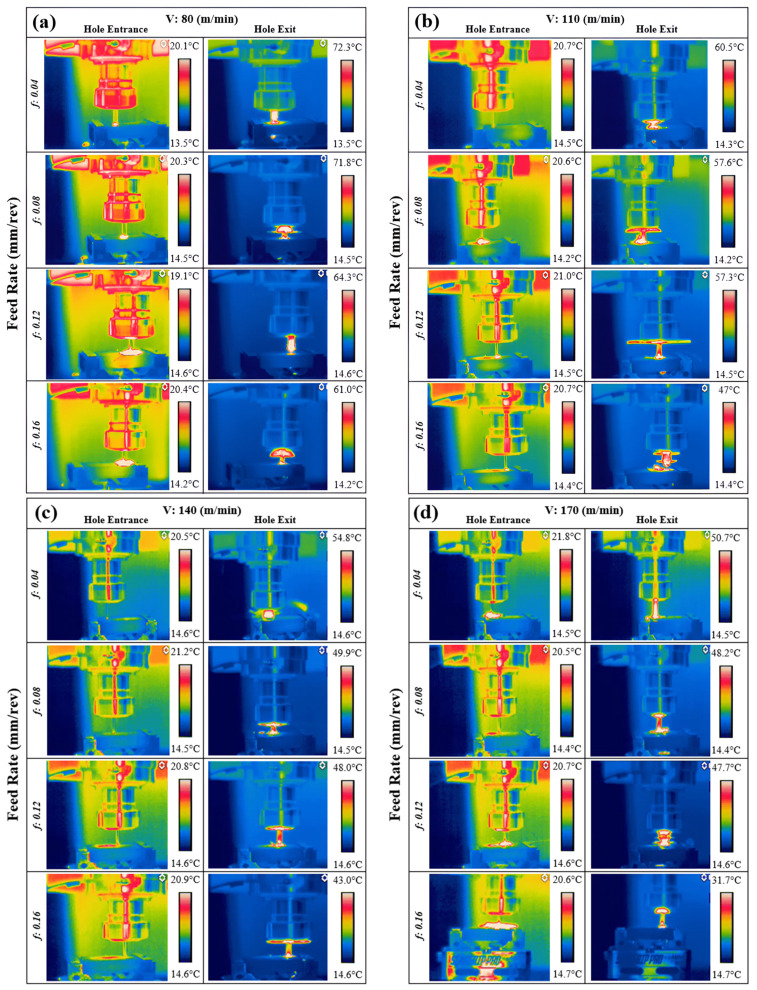
Infrared thermal images recorded at the hole entrance and hole exit during drilling of the hybrid PLA/17-4 PH/PLA structure under different *V* and *f* conditions: (**a**) 80 m/min, (**b**) 110 m/min, (**c**) 140 m/min, and (**d**) 170 m/min.

Thermal images show that the temperature range during drilling of the hybrid PLA/17-4 PH/PLA structure varies significantly depending on both the cutting parameters and the hole location (Figure 6a–d). The most striking finding is that while the temperatures measured at the hole entrance remained within a narrow range under all experimental conditions, much higher maximum temperatures were observed at the hole exit. Maximum temperatures at the hole entrance ranged from approximately 19.1 to 21.8 °C, while at the hole exit these values reached a range of 31.7 to 72.3 °C. The highest exit temperature was measured at 72.3 °C at a V of 80 m/min and an *f* of 0.04 mm/rev (Figure 6a), while the lowest value was determined as 31.7 °C at 170 m/min and 0.16 mm/rev (Figure 6d). Accordingly, it is observed that the maximum surface temperature recorded at the hole exit decreases with increasing *V* and *f* in the examined experimental range [64,65,66]. At a V of 80 m/min, the maximum hole entry temperatures ranged from 19.1 to 20.4 °C, while the maximum hole exit temperatures ranged from 61.0 to 72.3 °C (Figure 6a). Under this condition, the highest temperature was measured in the hole exit region at an *f* of 0.04 mm/rev. At a V of 110 m/min, the maximum hole entry temperatures ranged from 20.6 to 21.0 °C, and the maximum hole exit temperatures ranged from 47.0 to 60.5 °C (Figure 6b). At a V of 140 m/min, the maximum temperatures measured in the entry region remained between 20.5 and 21.2 °C, while the maximum temperatures recorded in the exit region ranged from 43.0 to 54.8 °C (Figure 6c). At a V of 170 m/min, the maximum hole entry temperatures ranged from 20.5 to 21.8 °C, while the maximum hole exit temperatures were measured in the range of 31.7 to 50.7 °C (Figure 6d). Overall, it is observed that the hole entry temperatures remained quite close to each other in all parameter combinations, while the hole exit temperatures were more sensitive to both *V* and *f*. Furthermore, it was determined that the maximum temperatures measured at the hole exit decreased regularly with increasing *f* at all *V*. This decrease in hole-exit temperature with increasing cutting speed and feed rate can be explained by the combined effects of reduced tool–workpiece contact time, shorter thermal residence near the exit region, and more rapid chip evacuation. Although higher material removal rates may locally increase instantaneous heat generation, the faster passage of the drill through the lower PLA layer limits the time available for heat accumulation at the exit surface. In addition, the heterogeneous PLA/17-4 PH/PLA architecture may restrict uniform heat diffusion through the specimen thickness. Therefore, the observed trend should be interpreted as a contact-time- and heat-residence-controlled thermal response rather than a simple reduction in heat generation.

The significant temperature concentration observed at the exit of the hole indicates that heat is not distributed homogeneously throughout the material during drilling and that the exit region becomes a critical thermal area. This trend is consistent with findings from drilling studies conducted using infrared thermography in composite materials. Fu et al. showed that exit temperatures are highly sensitive to material-tool interaction and that the location of maximum temperatures changes with drilling depth [64]. Similarly, Xu et al. reported that the maximum drilling temperature occurs in most cases when the tool cutting edges fully interact with the workpiece, and then the temperature rapidly decreases when the tool begins to exit the end layers [67]. In the present study, it is assessed that the heat generated by friction and plastic deformation during the drill’s progression from the top PLA layer to the middle 17-4 PH region and then to the bottom PLA layer accumulates, especially in the region near the exit phase; higher temperatures are recorded at the exit of the hole due to weakening of support conditions and increased local energy concentration.

The decrease in hole exit temperature as *V* and *f* increase, while seemingly different from the classical metal cutting approach at first glance, is a phenomenon encountered in composite drilling studies using infrared thermography. Khashaba and Alazwari emphasized that the temperature-parameter relationship in drilling is not unidirectional in the literature; it is closely related to the material system, tool geometry, laminate structure, and especially the measurement technique. In the same study, it was explicitly stated that instrumented drills record the tool tip temperature, while IR cameras record the surface temperature during drilling. Furthermore, it is stated that IR thermography cannot directly detect the internal thermal field but mainly shows the temperature distribution on the measured surface [65]. Therefore, the lower temperatures recorded in this study under high *V* and high *f* conditions may reflect not an absolute decrease in total heat generation, but rather a decrease in heat accumulation on the exit surface due to the shortening of the tool-workpiece contact time. In other words, although the amount of material removed per revolution increases with increasing *f*, the shortening of the time the tool spends in a particular region seems to have limited the observed thermal accumulation on the surface. This thermal behavior is thought to be related to the material architecture of the hybrid structure. In polymer-based structures produced by FDM, anisotropy, voids, and limited thermal conductivity lead to non-homogeneous heat transfer and the formation of local temperature zones. Shanmugam et al. reported that the thermal performance in FDM-printed polymeric materials is strongly affected by printing-induced anisotropy, void structure, and reinforcement type [68]. In this context, the different heat conduction and energy dissipation characteristics of the outer PLA layers and the central region containing 17-4 PH may have led to the uneven development of the thermal field across the layers during drilling. The significant temperature difference observed between the entry and exit regions in the present study is a result not only of the cutting parameters but also of the heterogeneous and multilayered character of the hybrid PLA/17-4 PH/PLA structure [69].

The maximum exit temperature of 72.3 °C measured under low *V* and low feed conditions is also important in terms of material behavior. A recent study reported that the glass transition temperature for 3D-printed PLA is approximately 65 °C. From this perspective, it is likely that the temperature value obtained, especially under the 80 m/min–0.04 mm/rev condition, triggered a local softening tendency in the PLA phase [66]. The study by Khashaba and Alazwari also showed that the surface temperature approaching or exceeding the critical temperature ranges of the polymer matrix can increase the risk of matrix softening and thermal degradation [65]. Therefore, the current thermal findings reveal that temperature is not only a process output but also a decisive indicator in terms of hole surface integrity, material smudging, and potential exit damage.

### 3.3. Vibration Response Under Different Drilling Conditions

The vibrational behavior generated during the drilling process was evaluated not only as an amplitude-based quantity but also in terms of frequency distribution. For this purpose, vibration signals were examined based on energy distribution in the frequency axis, and the spectral energy approach was adopted using the amplitude equation:

(2) $$E\left(k\right)={\left|A\left(k\right)\right|}^{2}\;$$

In the study, vibration data were evaluated in their raw form without normalization; RMS (Root Mean Square) was used for the total vibration level, $f_{peak}$ for the dominant frequency character, and $E_{total},\;E_{low},\;E_{middle}$ parameters were interpreted together for energy distribution. Furthermore, the $R_{high}=\;{\;E}_{high}/{\;E}_{low}$ parameter, representing the ratio of high-frequency components to low-frequency components, was considered as a relative indicator of the tendency towards unstable cutting. This approach reveals not only the magnitude of the vibration but also its structural and shear-induced dynamic characteristics.

The tooth traverse frequency limits were calculated considering a *V* range of 80–170 m/min, a drill diameter of 6 mm, and a double-edged tool; accordingly, the range of approximately 140–300 Hz was accepted as the characteristic cutting region. Based on these limits, the 0–300 Hz range was defined as the low frequency band, the 300–800 Hz range as the medium frequency band, and the 800 Hz and above range as the high frequency band. It was assumed that the low-frequency region is more related to cutting forces, the medium-frequency region to the overall dynamic response of the system, and the high-frequency region to unstable cutting, sudden energy concentrations, and local vibration disturbances. This classification is consistent with frequency-based approaches proposed in chatter and dynamic stability analyses in metal and composite machining processes [70,71,72,73,74].

As shown in Table 2, there are significant differences in vibration magnitude and energy distribution among the experiments. RMS values ranged from 0.03524 to 0.15651 m/s^2^, while the $f_{peak}$ values were approximately between 70.13 and 150.47 Hz. However, it does not seem possible to make a direct and sufficient interpretation about process stability or hole quality based solely on RMS levels. Indeed, while surface quality did not improve to the expected level in some experiments with low RMS values, more stable processing behavior was obtained in some experiments with medium RMS values. This situation indicates that vibration behavior should be evaluated not only based on total amplitude but also in conjunction with the energy distribution in the frequency bands.

In this context, the most striking comparison emerges between Experiment 4, which represents the worst results, and Experiment 13, which yields the most balanced results. According to Table 2, in Experiment 4, the RMS value was calculated as 0.14034 m/s^2^, $f_{peak}$ 100.08 Hz, $E_{total}$ 1.60 × 10^7^, $E_{low}$ 6.97 × 10^6^, $E_{middle}$ 5.75 × 10^6^, $E_{high}$ 3.24 × 10^6^ and $R_{high}$ 0.2030. In contrast, in Experiment 13, the RMS value was determined as 0.10195 m/s^2^, $f_{peak}$ 150.40 Hz, $E_{total}$ 1.66 × 10^7^, $E_{low}$ 1.15 × 10^7^, $E_{middle}$ 2.76 × 10^6^, $E_{high}$ 2.29 × 10^6^ and $R_{high}$ 0.1386. Remarkably, although the total energy values are close, the distribution of energy between the bands differs significantly. In Experiment 13, the energy is concentrated more in the low-frequency band, while in Experiment 4, the relative share of the medium and especially high-frequency components has increased. This indicates that Experiment 13 exhibits a more stable and predictable dynamic behavior, while Experiment 4 shows a more irregular and unstable vibrational structure.

Table 3, which evaluates the vibration data in conjunction with surface roughness and force-moment rankings, also supports this interpretation. Experiment 13 ranked 1st in both surface roughness and force-moment rankings, while remaining at only the moderate level in terms of RMS and $E_{high}$. In contrast, Experiment 4 ranked 16th in both surface roughness and force-moment rankings; its RMS and $E_{high}$ levels were classified as high. This result shows that the best drilling performance is obtained not at the minimum vibration level, but under conditions where high-frequency components are not dominant and the energy distribution is more balanced. In other words, rather than the absolute vibration amplitude, the frequency band in which the vibration energy is concentrated seems to be more decisive in terms of hole quality and drilling stability.

**Figure 7:**
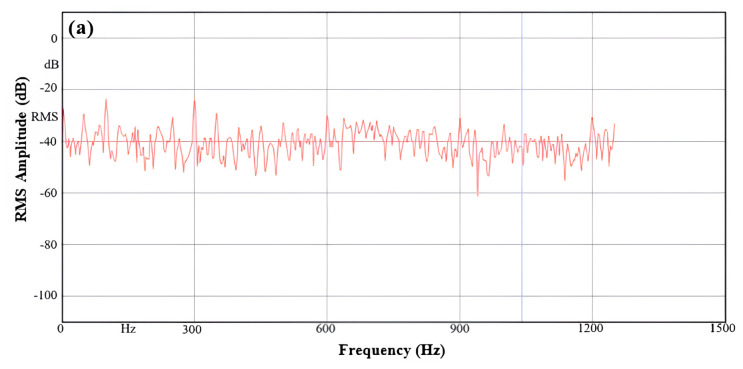

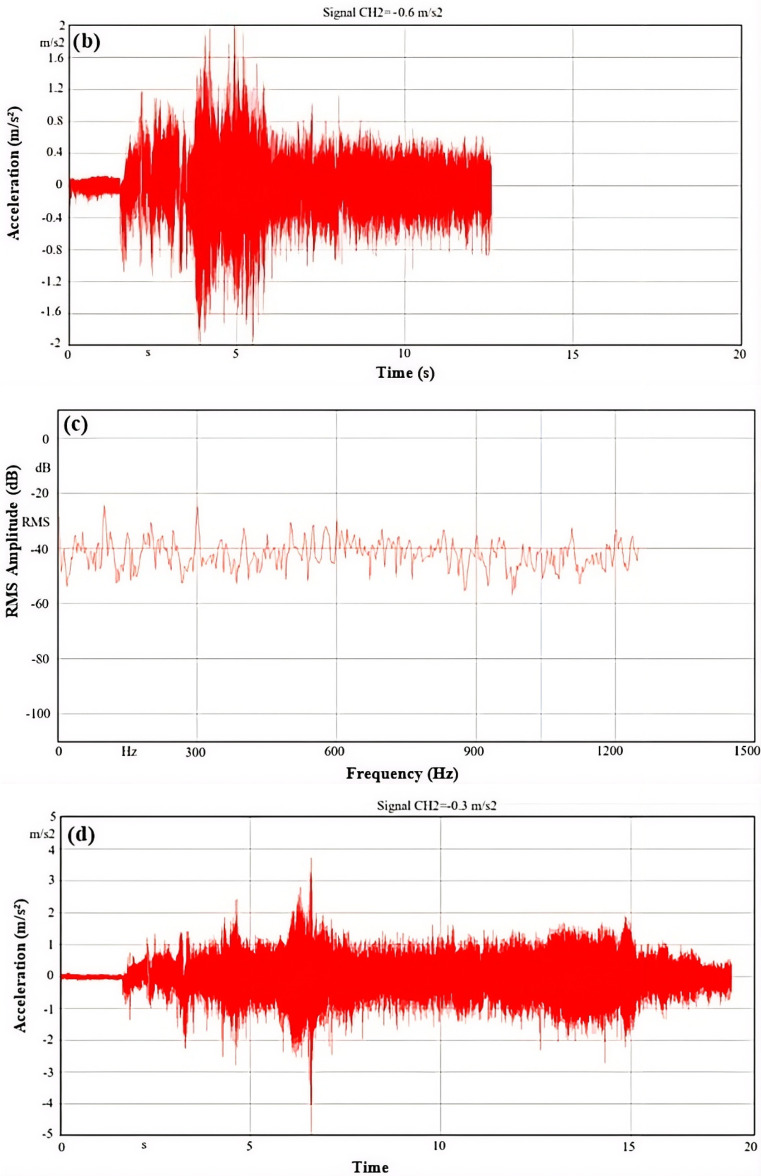
Comparison of the spectral and time-domain vibration responses obtained along the drilling axis for the selected drilling conditions: (**a**) FFT spectrum of Experiment 4, (**b**) time-domain vibration signal of Experiment 4, (**c**) FFT spectrum of Experiment 13, and (**d**) time-domain vibration signal of Experiment 13.

When the FFT and signal graphs of Experiment 4 are examined together with the corresponding spectral and temporal responses of Experiment 13, this difference can be observed more clearly (Figure 7). In Experiment 4, the signal amplitude shows a more irregular character with sudden spikes, and the high-frequency contribution is more pronounced in the FFT distribution. In contrast, in Experiment 13, although the vibration signal is not entirely low-level, the energy distribution is more controlled, and the relative dominance of high-frequency components is reduced. This observation is quantitatively supported by the decrease in the $R_{high}$ ratio. Therefore, the vibration response obtained along the drilling axis defines the optimum processing condition not only with the total amplitude magnitude but also with the limitation of high-frequency instability components.

When evaluated at the parameter level, the most optimal result was obtained at a *V* of 170 m/min and an *f* of 0.04 mm/rev; the most unfavorable result occurred at a *V* of 80 m/min and an *f* of 0.16 mm/rev, which is consistent with the trends determined in previous sections for force, moment, surface roughness, and temperature. The combination of low *V* and high *f* negatively affected drilling quality by creating higher mechanical load in the cutting zone, more irregular layer transitions, and more pronounced high-frequency vibration content. Conversely, the combination of high *V* and low *f* not only reduced force and moment levels but also prevented the dominance of high-frequency components, resulting in more balanced dynamic behavior. In this context, it was concluded that the optimum condition for drilling hybrid PLA/17-4 PH/PLA structures should be defined by a dynamic regime with balanced frequency distribution and limited high-frequency energy, rather than minimum vibration amplitude. In conclusion, while vibration data alone provides limited information, when interpreted together with force, moment, and surface roughness results, it becomes a powerful tool in explaining drilling performance. In particular, the $E_{high}$ and $R_{high}$ parameters stand out as distinctive indicators in monitoring unstable cutting components that can degrade surface quality. Therefore, it can be said that in evaluating drilling performance in hybrid structures, analyses based solely on total vibration magnitudes, such as RMS, are insufficient; an energy-based approach divided into frequency bands provides more explanatory and reliable results. These observations further support the correlation analysis, indicating that surface quality is more strongly associated with cutting load parameters (Fz and Mz) than with overall vibration magnitude, particularly when high-frequency components are not dominant.

### 3.4. Hole Surface Quality

Through-hole surface images show that the surface integrity of the hybrid PLA/17-4 PH/PLA structure after drilling is significantly sensitive to cutting parameters. When the images are evaluated generally, differences depending on the experimental conditions are revealed in terms of the continuity of machining traces along the hole wall, morphological irregularities in layer transition zones, local void formations, and the distribution of subsurface damage zones. In particular, the discontinuities observed in the interface regions between the PLA and 17-4 PH layers indicate that the tool-material interaction is not homogeneous throughout the drilling process due to the hybrid structure consisting of layers exhibiting different mechanical behaviors. From a mechanistic perspective, surface formation in the hybrid structure is governed by the combined effects of plastic deformation in the PLA layers, particle–matrix interaction in the metal-filled middle layer, and interfacial discontinuities between layers. In polymer regions, increased ductility and thermal softening promote material smearing and surface tearing, whereas in the metal-filled layer, the presence of rigid particles alters chip formation and leads to relatively smoother surfaces. At the layer transition zones, abrupt changes in stiffness and cutting resistance contribute to local instability, resulting in micro-voids, irregular tool engagement, and surface discontinuities. It is known that such internal surface irregularities are related to layered architecture, interstitial discontinuities, and thermal-mechanical loads generated during machining in additively manufactured polymer-based structures [61,62].

**Figure 8:**
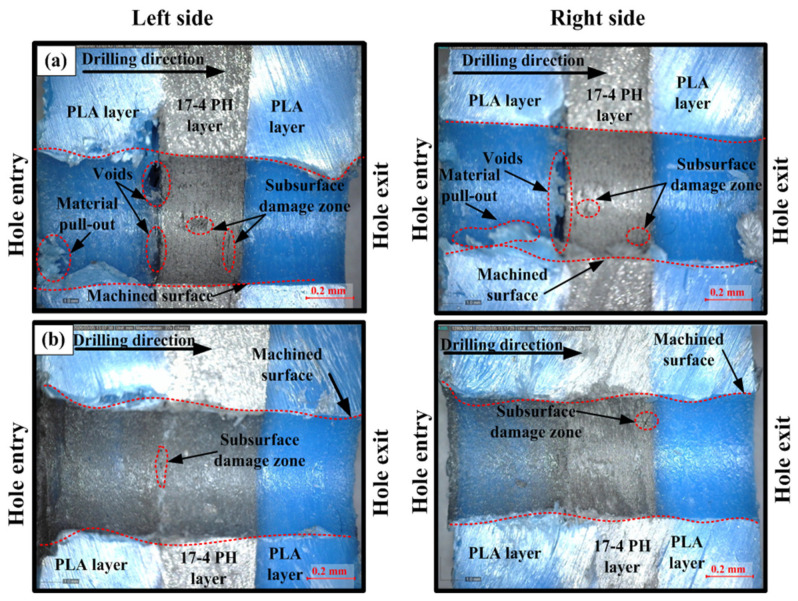
Inner hole surface images of the hybrid PLA/17-4 PH/PLA structure after drilling under two cutting conditions: (**a**) 80 m/min, 0.16 mm/rev; (**b**) 170 m/min, 0.04 mm/rev.

In test condition number 4 (80 m/min, 0.16 mm/rev), representing the worst drilling performance, more pronounced machining marks, localized void concentrations, and larger subsurface damage areas were observed in regions near the interface (Figure 8a). This appearance, when evaluated together with the high thrust force, moment, and surface roughness values measured under the same condition, suggests that the combination of low *V* and high *f* creates a more aggressive material removal regime in the hole wall. The literature also reports that increasing the *f* increases the thrust force, resulting in more pronounced deformations around and inside the hole; however, more stable drilling behavior is obtained under suitable cutting conditions. It has been reported that, especially in PLA-based parts produced with FFF/FDM, increasing the *f* increases the force levels, and high mechanical loads negatively affect hole quality [44,48]. In contrast, under test condition 13 (170 m/min, 0.04 mm/rev), which represents the best drilling performance, the inner surface of the hole exhibited a smoother, more homogeneous, and better-preserved continuity (Figure 8b). Under this condition, it is understood that the machining traces are more regular, the formation of voids is more limited, and subsurface damage areas are represented by narrower areas. The combination of higher *V* and lower *f* contributed to a reduction in mechanical loads by making the tool-workpiece contact more stable; this enabled a more controlled surface formation on the hole wall. Indeed, current studies report that low *f*s and high *V*s are associated with lower Fz and Mz values and that this improves the hole surface morphology and overall hole quality. It has also been shown that lower mechanical loads in PLA and similar additively manufactured thermoplastic structures improve quality indicators such as surface roughness, delamination, circularity, and cylindricity [62,75].

Overall, the through-hole surface morphology findings confirmed the mechanical drilling performance results. While more pronounced surface irregularities, localized voids, and damage-affected areas were observed in test condition 4, a more homogeneous machined surface and more limited morphological distortions were obtained in test condition 13. These findings demonstrate that the combination of high V and low f provides better surface integrity in the hybrid PLA/17-4 PH/PLA structure. Nevertheless, these observations should be interpreted as internal comparisons among the tested drilling conditions of the hybrid structure. Since a monolithic PLA control was not drilled in the present study, the current surface findings cannot be used to claim superior hole quality relative to pure PLA.

### 3.5. Principal Component Analysis of Drilling Responses

To better understand the interrelationships among the measured drilling outputs and to identify the dominant factors governing the overall machining behavior of the hybrid PLA/17-4 PH/PLA structure, Principal Component Analysis (PCA) was performed using the layer-based thrust force (Fz), torque (Mz), and surface roughness (Ra) values together with Tmax and the high-frequency vibration component (E_high). According to the PCA results, the first principal component (PC1) had an eigenvalue of 7.63 and explained 69.4% of the total variance, whereas the second principal component (PC2) had an eigenvalue of 1.58 and explained an additional 14.3%. Thus, the first two principal components accounted for 83.7% of the total variance, indicating that the multidimensional dataset can be effectively interpreted within a two-component PCA space. The eigenvalues and explained variance ratios of the retained components are summarized in Table 4. Based on the Kaiser criterion (eigenvalue > 1), only PC1 and PC2 were retained for further analysis.

**Figure 9:**
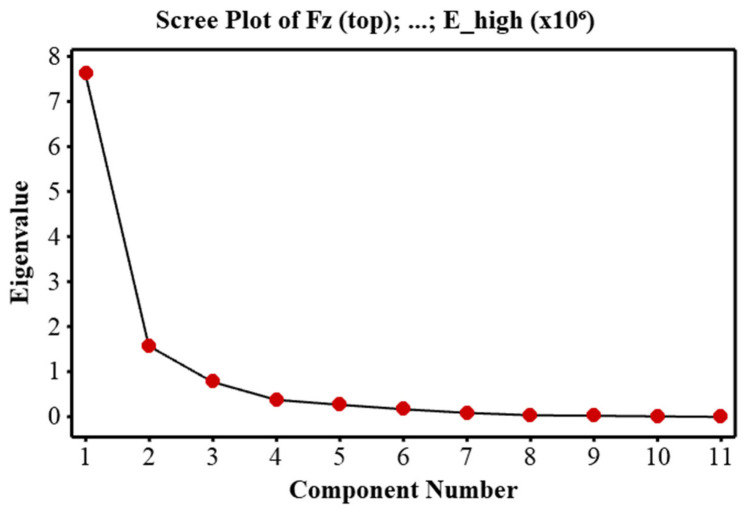
Scree plot of the principal components showing that PC1 and PC2 capture most of the variance.

The scree plot also supported this selection by showing a sharp decline after the second component and a clear flattening trend for the remaining components (Figure 9). This behavior indicates that most of the meaningful variation in the dataset is concentrated in the first two components, while the contribution of the remaining components is limited. Therefore, the retained two-component structure can be considered sufficient to interpret the dominant response patterns of the drilling process in the studied hybrid architecture.

**Figure 10:**
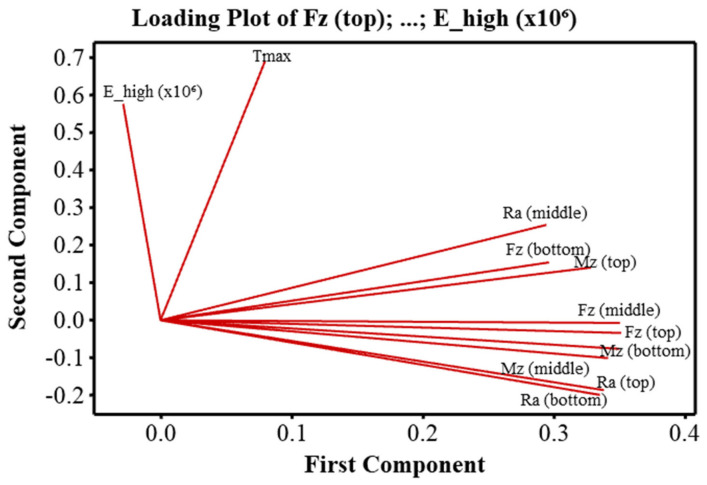
Loading plot of PC1 and PC2 showing the relationships among force, torque, surface roughness, temperature, and vibration variables.

The loading plot revealed that the layer-based Fz, Mz, and Ra variables were grouped predominantly along PC1 with consistently positive loadings (Figure 10). This indicates that PC1 mainly represents the mechanical load–surface quality axis of the drilling process. In practical terms, this means that the increase in thrust force and torque is directly accompanied by a deterioration in surface quality across the top, middle, and bottom layers. This result is fully consistent with the findings presented in Section 3.1 and Section 3.4, where unfavorable cutting conditions were characterized by simultaneously higher force, moment, and roughness values together with more pronounced internal surface irregularities. Therefore, PCA statistically confirms that the degradation of hole quality in the studied hybrid structure is governed primarily by mechanical loading conditions. From a physical viewpoint, the dominance of PC1 suggests that the hybrid structure responds first and foremost through load-controlled machining behavior. This can be attributed to the heterogeneous layer architecture of the PLA/17-4 PH/PLA configuration, where differences in local stiffness, deformation resistance, and interfacial interaction directly influence cutting resistance. As the drill passes through the layered structure, the resulting variations in force and torque are reflected almost simultaneously in the generated surface roughness. The fact that Fz, Mz, and Ra remain strongly grouped within the same principal component shows that these responses are not independent, but instead form a coupled mechanical–surface integrity domain.

In contrast, PC2 was mainly dominated by Tmax and E_high, indicating that the second component represents the thermal and dynamic response domain of the drilling process (Figure 10). The separation of Tmax and E_high from the force–moment–roughness cluster suggests that thermal accumulation and high-frequency vibration content contribute to process variability through a secondary but clearly distinguishable mechanism. This is an important outcome because it shows that temperature rise and unstable vibration content do not act as the primary determinants of drilling quality on their own but rather modify the response generated by the main mechanical load domain. In this sense, the PCA findings support the interpretation made in Section 3.2 and Section 3.3 that thermal effects and vibration behavior should be evaluated as complementary response indicators rather than isolated governing variables.

**Figure 11:**
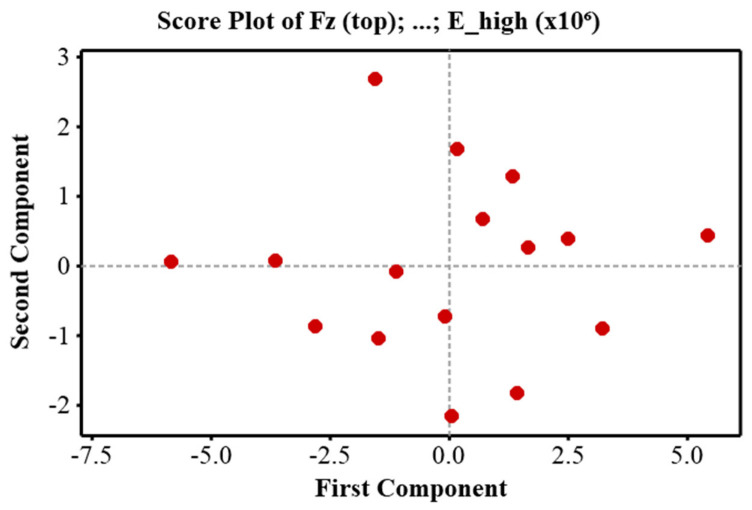
Score plot of PC1 and PC2 illustrating the distribution of the experimental conditions.

The score plot further showed that the experimental conditions were distributed differently within the PC1–PC2 space (Figure 11), confirming that different cutting parameter combinations produced distinct response patterns. Conditions associated with higher cutting loads and poorer surface quality tended to shift toward the positive side of PC1, whereas more favorable drilling conditions were positioned in regions characterized by lower mechanical loading and more balanced thermal–dynamic behavior. This clustering behavior demonstrates that the combined evaluation of force, torque, roughness, temperature, and vibration provides a more comprehensive interpretation of drilling performance than considering each response separately. It also indicates that the most favorable drilling condition is not defined by a single minimum response value but by a balanced combination of reduced mechanical loading and controlled thermal–dynamic effects.

Overall, the PCA results provide multivariate support for the findings obtained from the individual analyses presented in Section 3.1, Section 3.2, Section 3.3 and Section 3.4. More importantly, beyond simply confirming these trends, the PCA clarifies the hierarchical structure of the drilling responses in the hybrid PLA/17-4 PH/PLA system. The machining behavior is governed primarily by a dominant mechanical–surface integrity domain, while thermal effects and high-frequency vibration components act as secondary modifiers of that response. This distinction is valuable for interpreting hybrid drilling behavior because it shows that optimization of the process should first focus on reducing mechanical load and preserving surface integrity, while thermal and dynamic control should be considered as supportive refinements. Therefore, the PCA not only validates the experimental observations but also strengthens the overall interpretation of the machinability of the layered hybrid structure.

### 3.6. Generalized Findings, Practical Recommendations, and Future Perspectives

In order to provide a more integrated interpretation of the drilling behavior of the hybrid PLA/17-4 PH/PLA structure, the main experimental findings were comparatively evaluated in terms of mechanical loading, surface quality, thermal response, and vibration behavior. The obtained results revealed that the drilling performance of the studied green-state hybrid configuration was strongly governed by the combined influence of cutting speed and feed rate. In general, increasing the feed rate intensified thrust force, torque, and surface roughness, whereas increasing the cutting speed promoted a more stable drilling regime with lower mechanical loading and reduced thermal accumulation. A generalized summary of the main findings, practical implications, and recommended drilling conditions is presented in Table 5.

Based on the overall evaluation of the drilling outputs, the parameter combination of 170 m/min and 0.04 mm/rev can be recommended as the most favorable drilling condition within the investigated range, particularly for applications requiring lower cutting load, improved hole-wall quality, and more stable dynamic behavior. In contrast, the combination of 80 m/min and 0.16 mm/rev resulted in the least favorable drilling response due to increased force generation, higher roughness, and less stable cutting behavior. These findings indicate that the simultaneous control of cutting speed and feed rate is critically important for preserving the surface integrity and machining stability of layered hybrid structures produced by FDM.

## 4. Conclusions

In this study, the drilling behavior of an FDM-printed green-state PLA/17-4 PH metal-filled/PLA layered structure was evaluated in terms of thrust force, torque, surface roughness, temperature variation, vibration response, and internal hole surface integrity. The main conclusions are as follows:

- Drilling performance was strongly governed by cutting speed, feed rate, and the heterogeneous layered architecture. Increasing the feed rate increased thrust force, torque, and surface roughness, whereas increasing the cutting speed generally reduced these responses and promoted a more stable drilling regime.
- The most favorable drilling condition was obtained at 170 m/min and 0.04 mm/rev, while the least favorable condition was observed at 80 m/min and 0.16 mm/rev. Under the best condition, the middle 17-4 PH-containing layer showed reductions of approximately 41.8% in thrust force, 56.5% in torque, and 25.6% in surface roughness compared with the worst condition.
- Layer-based evaluations showed that the middle 17-4 PH-containing layer had the highest cutting resistance, with Fz values ranging from 10.05 to 17.28 N and Mz values from 7.19 to 16.53 N·cm. In contrast, the PLA layers exhibited higher surface roughness, with Ra values of 12.8754–15.4465 µm in the top layer and 11.993–14.2962 µm in the bottom layer.
- Thermal analysis showed that the hole-exit region was more critical than the hole-entry region. While maximum temperatures at the hole entry remained within a narrow range of 19.1–21.8 °C, the hole-exit temperatures varied more widely between 31.7 and 72.3 °C, indicating localized thermal accumulation near the exit zone.
- Vibration results indicated that total vibration amplitude alone was insufficient to explain drilling quality. High-frequency vibration components were more useful for identifying unstable cutting behavior and should therefore be evaluated together with force, torque, roughness, and temperature responses.
- From a practical perspective, high cutting speed combined with low feed rate can be recommended as an effective drilling strategy for green-state PLA/17-4 PH/PLA hybrid FDM parts. This parameter window can help improve hole quality, reduce cutting load, limit unstable vibration behavior, and enhance post-processing reliability.
- The study does not claim that the hybrid structure is mechanically superior to monolithic PLA or that the green-state 17-4 PH-containing layer behaves as a fully metallic core. Rather, it clarifies the drilling response of a heterogeneous as-printed layered structure and provides guidance for optimizing secondary machining operations of multi-material FDM components.
- The focus on the green-state configuration is particularly important because such structures may undergo secondary machining prior to debinding or sintering during prototyping and hybrid manufacturing processes. In addition, similar polymer/metal-filled/polymer sandwich architectures may have potential functional relevance for localized mass modification, magnetic response, electromagnetic interference shielding, thermal moderation, and functionally graded prototyping; however, these applications require dedicated experimental validation in future studies.

Overall, these findings provide practical guidance for selecting drilling parameters in the post-processing of additively manufactured multi-material structures. In particular, the results demonstrate that reliable hole quality in green-state PLA/17-4 PH/PLA hybrid parts requires the simultaneous control of mechanical loading, thermal accumulation, vibration stability, and surface integrity. Future studies should further investigate different tool geometries, coated drills, cooling or lubrication strategies, alternative layer configurations, and multi-objective optimization approaches to improve the machinability of hybrid FDM structures.

## Figures and Tables

**Table 1:** Technical properties and printing parameters for the filaments.

Property	PLA	17-4 PH Stainless Steel-Based Filament (Ultrafuse^®^)
Company	eSUN	BASF Forward AM
Filament diameter (mm)	1.75	1.75
Density (g/cm^3^)	1.2	7.6
Tensile strength (MPa)	72	990
Elongation to fracture (%)	11.8	4
Printing temperature (°C)	210–260	230–250
Bed temperature (°C)	45–60	90–100
Printing speed (mm/s)	40–100	15–70

Note: The listed nominal properties of the 17-4 PH material are based on supplier information and do not represent the effective mechanical properties of the as-printed green-state middle layer used in this study.

**Table 2:** Vibration values along the drilling axis.

Exp. No.	RMS (m/s^2^)	$f_{peak}$(Hz)	$E_{total}$	$E_{low}$	$E_{middle}$	$E_{high}$	$R_{high}$
1	0.06654	70.13	7.80 × 10^6^	5.82 × 10^6^	1.28 × 10^6^	6.93 × 10^5^	0.0889
2	0.12113	100.07	3.33 × 10^7^	1.82 × 10^7^	9.80 × 10^6^	5.20 × 10^6^	0.1562
3	0.10817	99.97	1.98 × 10^7^	9.57 × 10^6^	6.39 × 10^6^	3.86 × 10^6^	0.1949
**4**	**0.14034**	**100.08**	**1.60 × 10^7^**	**6.97 × 10^6^**	**5.75 × 10^6^**	**3.24 × 10^6^**	**0.2030**
5	0.14761	97.43	9.61 × 10^7^	5.44 × 10^7^	2.03 × 10^7^	2.14 × 10^7^	0.2228
6	0.06055	99.95	2.76 × 10^7^	1.62 × 10^7^	6.41 × 10^6^	5.03 × 10^6^	0.1819
7	0.12557	98.49	1.63 × 10^7^	8.68 × 10^6^	3.10 × 10^6^	4.51 × 10^6^	0.2766
8	0.15651	79.09	1.58 × 10^7^	1.07 × 10^7^	2.83 × 10^6^	2.20 × 10^6^	0.1395
9	0.09236	123.74	1.44 × 10^7^	7.05 × 10^6^	5.84 × 10^6^	1.47 × 10^6^	0.1024
10	0.04656	100.04	6.00 × 10^7^	2.34 × 10^7^	2.99 × 10^7^	6.79 × 10^6^	0.1132
11	0.11494	100.10	1.54 × 10^7^	6.83 × 10^6^	6.44 × 10^6^	2.14 × 10^6^	0.1391
12	0.03524	124.11	1.21 × 10^6^	7.74 × 10^5^	3.31 × 10^5^	1.00 × 10^5^	0.0832
**13**	**0.10195**	**150.40**	**1.66 × 10^7^**	**1.15 × 10^7^**	**2.76 × 10^6^**	**2.29 × 10^6^**	**0.1386**
14	0.15394	150.42	2.89 × 10^7^	2.20 × 10^7^	4.23 × 10^6^	2.69 × 10^6^	0.0930
15	0.13104	150.46	2.02 × 10^7^	1.58 × 10^7^	2.73 × 10^6^	1.72 × 10^6^	0.0851
16	0.12646	150.47	4.98 × 10^7^	3.90 × 10^7^	6.50 × 10^6^	4.29 × 10^6^	0.0863

Note: Bold values indicate the minimum and maximum values within the corresponding columns.

**Table 3:** Relative values of force, moment, surface roughness, and vibration along the drilling axis.

Exp. No.	Surface Roughness Ranking	Force and Moment Ranking	RMS (m/s^2^)	$E_{high}$
1	7	8	Low	Very low
2	9	12	Medium	Medium
3	13	15	Medium	High
**4**	**16**	**16**	**High**	**High**
5	5	4	High	High
6	10	10	Low	High
7	14	13	High	Very High
8	15	14	High	Medium
9	2	3	Medium	Low
10	4	6	Low	Low
11	8	7	Medium	Medium
12	12	11	Very low	Very low
**13**	**1**	**1**	**Medium**	**Medium**
14	3	2	High	Low
15	6	5	Medium	Low
16	11	9	Medium	Medium

Note: Bold values indicate the best and worst-ranked experimental conditions in the table.

**Table 4:** Eigenvalues and explained variance ratios of the retained principal components (PC1 and PC2).

Principal Component	Eigenvalue	Variance (%)	Cumulative (%)
PC1	7.630	69.4	69.4
PC2	1.577	14.3	83.7

**Table 5:** Generalized summary of the main experimental findings and practical drilling recommendations for the hybrid PLA/17-4 PH/PLA structure.

Performance Indicator	Main Observed Trend	Practical Implication	Recommended Drilling Condition
Thrust force (Fz)	Increased with feed rate and decreased with cutting speed	Lower thrust force reduces mechanical loading and interfacial instability during drilling	High cutting speed and low feed rate
Torque (Mz)	Increased with feed rate, especially in the 17-4 PH-containing middle layer	Lower torque supports more stable material removal in the heterogeneous drilling path	High cutting speed and low feed rate
Surface roughness (Ra)	Increased with feed rate; PLA layers showed higher Ra than the middle layer	Low feed rate is essential for improving hole-wall quality and reducing surface tearing	170 m/min and 0.04 mm/rev
Temperature	Hole-entry temperature remained limited, whereas hole-exit temperature was more sensitive to cutting conditions	Thermal accumulation should be controlled, especially near the exit region	Higher cutting speed with controlled feed
Vibration response	Overall vibration level alone was insufficient; high-frequency components better reflected unstable cutting behavior	Frequency-domain vibration analysis should accompany force and roughness measurements	Conditions with reduced high-frequency energy
Overall drilling performance	Best performance was obtained at 170 m/min and 0.04 mm/rev; worst performance at 80 m/min and 0.16 mm/rev	Optimal drilling requires simultaneous control of load, surface quality, thermal response, and vibration stability	170 m/min and 0.04 mm/rev

## Data Availability

The original contributions presented in the study are included in the article. Further inquiries can be directed to the corresponding author.

## References

[1] Alparslan C., Bayraktar Ş. (2025). Advances in digital light processing (DLP) bioprinting: A review of biomaterials and its applications, innovations, challenges, and future perspectives. Polymers.

[2] Alparslan C., Bayraktar Ş. (2026). Additive manufacturing in biomaterials: A comprehensive and integrated review of innovations in tissue engineering, bioprinting, implant design, and regenerative medicine. Surf. Topogr. Metrol. Prop..

[3] Cano-Vicent A., Tambuwala M.M., Hassan S.S., Barh D., Aljabali A.A., Birkett M. (2021). Fused deposition modelling: Current status, methodology, applications and future prospects. Addit. Manuf..

[4] García-Cabezón C., Naranjo J.A., García-Hernández C., Berges C., Herranz G., Martín-Pedrosa F. (2024). Using fused filament fabrication to improve the tribocorrosion behaviour of 17-4 PH SS in comparison to other metal forming techniques. Friction.

[5] Alparslan C., Bayraktar Ş., Gupta K. (2024). A comparative study on mechanical performance of PLA, ABS, and CF materials fabricated by fused deposition modeling. Facta Univ. Ser. Mech. Eng..

[6] Lalegani Dezaki M., Mohd Ariffin M.K.A., Hatami S. (2021). An overview of fused deposition modelling (FDM): Research, development and process optimisation. Rapid Prototyp. J..

[7] Risad R.H., Ahmed M.H., Basher A., Rashid S., Shishir M.M.A., Hossain K.R. (2024). FDM printing process and its biomedical application. Chem. Res. Technol..

[8] Kantaros A., Katsantoni M., Ganetsos T., Petrescu N. (2025). The evolution of thermoplastic raw materials in high-speed FFF/FDM 3D printing era: Challenges and opportunities. Materials.

[9] Alparslan C., Şentürk E., Bayraktar Ş. (2026). Recent trends on additive manufactured advanced materials: A targeted review on functional capabilities and process integration. Surf. Topogr. Metrol. Prop..

[10] Ahangar P., Cooke M.E., Weber M.H., Rosenzweig D.H. (2019). Current biomedical applications of 3D printing and additive manufacturing. Appl. Sci..

[11] Stepashkin A.A., Chukov D.I., Senatov F.S., Salimon A.I., Korsunsky A.M., Kaloshkin S.D. (2018). 3D-printed PEEK-carbon fiber (CF) composites: Structure and thermal properties. Compos. Sci. Technol..

[12] Ventola C.L. (2014). Medical applications for 3D printing: Current and projected uses. Pharm. Ther..

[13] Nachal N., Moses J.A., Karthik P., Anandharamakrishnan C. (2019). Applications of 3D printing in food processing. Food Eng. Rev..

[14] Rane K., Strano M. (2019). A comprehensive review of extrusion-based additive manufacturing processes for rapid production of metallic and ceramic parts. Adv. Manuf..

[15] Su T.Y.T., Aguiar R., Sansone N.D., Hao C., Yan N., Lee P.C. (2025). Green, sustainable, and melt-compoundable PLA composites reinforced with spray-dried lignocellulosic nanofibrils for enhanced barrier and mechanical properties. Chem. Eng. J..

[16] Özkül M., Kuncan F., Ulkir O. (2025). Predicting mechanical properties of FDM-produced parts using machine learning approaches. J. Appl. Polym. Sci..

[17] Yuan Q., Kim M., Tien T.T., Fang Y., Duan Z., Zhang Y., Suhr J. (2025). Investigation of contour-related parameters’ effects on anisotropic mechanical properties and surface roughness of FDM-printed parts. Int. J. Adv. Manuf. Technol..

[18] Basak A.K., Sali J.M., Pramanik A. (2025). Mechanical properties of 17-4 PH stainless steel manufactured by atomic diffusion additive manufacturing. Designs.

[19] Singh D., Babbar A., Jain V., Gupta D., Saxena S., Dwibedi V. (2019). Synthesis, characterization, and bioactivity investigation of biomimetic biodegradable PLA scaffold fabricated by fused filament fabrication process. J. Braz. Soc. Mech. Sci. Eng..

[20] Mazur J., Sobczak P., Panasiewicz M., Łusiak P., Krajewska M., Findura P. (2025). Mechanical properties and biodegradability of samples obtained by 3D printing using FDM technology from PLA filament with by-products. Sci. Rep..

[21] Tümer E.H., Erbil H.Y. (2021). Extrusion-based 3D printing applications of PLA composites: A review. Coatings.

[22] Sandanamsamy L., Harun W.S.W., Ishak I., Romlay F.R.M., Kadirgama K., Ramasamy D. (2023). A comprehensive review on fused deposition modelling of polylactic acid. Prog. Addit. Manuf..

[23] Kalva S.N., Ali F., Velasquez C.A., Koç M. (2023). 3D-printable PLA/Mg composite filaments for potential bone tissue engineering applications. Polymers.

[24] Cojocaru V., Frunzaverde D., Miclosina C.O., Marginean G. (2022). The influence of the process parameters on the mechanical properties of PLA specimens produced by fused filament fabrication—A review. Polymers.

[25] Arockiam A.J., Subramanian K., Padmanabhan R.G., Selvaraj R., Bagal D.K., Rajesh S. (2022). A review on PLA with different fillers used as a filament in 3D printing. Mater. Today Proc..

[26] Orellana Barrasa J., Ferrández-Montero A., Ferrari B., Pastor J.Y. (2021). Characterisation and modelling of PLA filaments and evolution with time. Polymers.

[27] Nugraha A.D., Adi R.K., Kumar V.V., Kusumawanto A., Prawara B., Junianto E. (2024). Investigating the mechanical properties and crashworthiness of hybrid PLA/GFRP composites fabricated using FDM-filament winding. Heliyon.

[28] Yohannan A., Vincent S., Divakaran N., Pottikadavath Venugopal A.K., Patra S., Ashish K., Mohanty S. (2024). Experimental and simulation studies of hybrid MWCNT/montmorillonite reinforced FDM based PLA filaments with multifunctional properties enhancement. Polym. Compos..

[29] Wei X., Behm I., Winkler T., Scharf S., Li X., Bähr R. (2022). Experimental study on metal parts under variable 3D printing and sintering orientations using bronze/PLA hybrid filament coupled with fused filament fabrication. Materials.

[30] Manola M.S., Singh B., Singla M.K., Kumar R. (2023). Investigation of melt flow index of dual metal reinforced ABS polymer for FDM filament fabrication. Mater. Today Proc..

[31] Vakharia V.S., Kuentz L., Salem A., Halbig M.C., Salem J.A., Singh M. (2021). Additive manufacturing and characterization of metal particulate reinforced polylactic acid (PLA) polymer composites. Polymers.

[32] Naim M., Chemkhi M., Alhussein A., Retraint D. (2023). Effect of post-treatments on the tribological and corrosion behavior of 17-4 PH stainless steel processed via fused filament fabrication. Addit. Manuf. Lett..

[33] Caballero A., Ding J., Ganguly S., Williams S. (2019). Wire + Arc Additive Manufacture of 17-4 PH stainless steel: Effect of different processing conditions on microstructure, hardness, and tensile strength. J. Mater. Process. Technol..

[34] Liew Y., Tan A., Salehi M. (2025). Microstructural, mechanical, and electrochemical corrosion properties of extrusion additive manufactured 17-4 precipitation hardenable stainless steel. J. Mater. Eng. Perform..

[35] Ötüken R., Alparslan C., Erhan M.F., Bayraktar Ş. (2026). Reinforcement of novel PLA/17-4 PH stainless steel hybrid structures fabricated by FDM: The effects of layer configuration, infill density and pattern. Polymers.

[36] Mazeeva A., Masaylo D., Konov G., Popovich A. (2024). Multi-metal additive manufacturing by extrusion-based 3D printing for structural applications: A review. Metals.

[37] Zhang W., Li R., Peng Y., Xu H. (2024). Fused deposition modeling (FDM) based additive manufacturing of 17-4 PH stainless steel: Static and fatigue mechanical performance. SSRN.

[38] Amuthakkannan P., Manikandan V. (2025). Metal 3D printing—A comprehensive review on materials, methods and properties. J. Res. Appl. Mech. Eng..

[39] Mandpe K., Shrivas S.P., Thakur A. (2019). Performance analysis of various characteristics on dry drilling hole quality of 17-4 PH stainless steel with solid carbide drill bits. CSVTU Res. J..

[40] Naveen Anthuvan R., Hari Chealvan S., Krishnaraj V., Xu J. (2025). Exploring micro-machining performance: Drilling versus EDM for high-quality micro-holes in AM 17-4 PH stainless steel. Proc. Inst. Mech. Eng. Part E J. Process Mech. Eng..

[41] Kumar R., Hynes N.R.J. (2019). Thermal drilling processing on sheet metals: A review. Int. J. Lightweight Mater. Manuf..

[42] Ramaswamy A., Perumal A.V. (2020). Multi-objective optimization of drilling EDM process parameters of LM13 Al alloy–10ZrB2–5TiC hybrid composite using RSM. J. Braz. Soc. Mech. Sci. Eng..

[43] Rajmohan T., Palanikumar K. (2013). Application of the central composite design in optimization of machining parameters in drilling hybrid metal matrix composites. Measurement.

[44] Uşun A., Alparslan C., Erhan M.F., Kuleyin H., Gümrük R., Bayraktar Ş. (2026). Drilling performance evaluation of additively manufactured continuous carbon fiber reinforced thermoplastic composites. Polymers.

[45] Sneha N., Balamurugan K. (2022). Micro-drilling optimization study using RSM on PLA-bronze composite filament printed using FDM. Proceedings of the 2022 IEEE 2nd Mysore Sub Section International Conference (MysuruCon).

[46] Vishwadarshan, Shetty G., Shetty R., Supriya J.P., Balaji V., Hegde A. (2025). Comprehensive analysis of drilling responses in additively manufactured PLA using a regression-based statistical learning approach. Mater. Res. Express.

[47] Shunmugesh K., Sarker B., Chakraborty S. (2025). Parametric analysis, optimization and machine learning-based prediction during drilling of 3D-printed polylactic acid polymer. JMST Adv..

[48] Esiyok A., Ergene B. (2025). Drillability of 3D printed PLA parts: Influence of carbon fiber reinforcement, feed rate, cutting speed, and drill diameter. Polym. Adv. Technol..

[49] Madhan Kumar A., Jayakumar K. (2022). Mechanical and drilling characterization of biodegradable PLA particulate green composites. J. Chin. Inst. Eng..

[50] Masannan V., Anbalagan C., Lakshmaiya N., Kumar P. (2024). Experimental investigation on the drilling characteristics of kenaf/PLA-based laminates. Eng. Proc..

[51] eSUN. https://www.esun3d.com/tr/.

[52] BASF Forward AM, Ultrafuse 17-4 PH. https://forward-am.com/material-portfolio/ultrafuse-filaments-for-fused-filaments-fabrication-fff/metal-filaments/ultrafuse-17-4-ph/.

[53] Flashforge Creator 3 3D Yazıcı. https://www.flashforge.com.hk/creator-3.html.

[54] (2016). Parallel Shank Jobber and Stub Series Drills and Morse Taper Shank Drills.

[55] Lalegani Dezaki M., Mohd Ariffin M.K.A., Baharuddin B.T.H.T. (2021). Experimental study of drilling 3D printed polylactic acid (PLA) in FDM process. Fused Deposition Modeling Based 3D Printing.

[56] (1997). Geometrical Product Specifications (GPS)—Surface Texture: Profile Method—Terms, Definitions and Surface Texture Parameters.

[57] Lotfi A., Li H., Dao D.V. (2020). Drilling behavior of flax/poly (lactic acid) bio-composite laminates: An experimental investigation. J. Nat. Fibers.

[58] Ramesh B., Elayaperumal A., Satishkumar S., Kumar A., Jayakumar T. (2016). Effect of drill point geometry on quality characteristics and multiple performance optimization in drilling of nonlaminated composites. Proc. Inst. Mech. Eng. Part L J. Mater. Des. Appl..

[59] John R., Lin R., Jayaraman K., Bhattacharyya D. (2021). Effects of machining parameters on surface quality of composites reinforced with natural fibers. Mater. Manuf. Process..

[60] Shanmugam V., Marimuthu U., Rajendran S., Veerasimman A., Basha A.M., Majid M.S.B.A. (2021). Experimental investigation of thrust force, delamination and surface roughness in drilling hybrid structural composites. Materials.

[61] Baraheni M., Shabgard M.R., Amini S. (2021). Evaluating the hole quality produced by vibratory drilling: Additive manufactured PLA+. Int. J. Adv. Manuf. Technol..

[62] Shunmugesh K., Ganesh M., Bhavani R., Khan M.A., Saravana Kumar M., Rajeshkumar L. (2025). Enhancing drilling performance in 3D printed PLA implants application of PIV and ML models. Sci. Rep..

[63] Bayraktar Ş., Alparslan C., Salihoğlu N., Sarıkaya M. (2025). A holistic research based on RSM and ANN for improving drilling outcomes in Al–Si–Cu–Mg (C355) alloy. J. Mater. Res. Technol..

[64] Fu R., Jia Z., Wang F., Jin Y., Sun D., Yang L., Cheng D. (2018). Drill-exit temperature characteristics in drilling of UD and MD CFRP composites based on infrared thermography. Int. J. Mach. Tools Manuf..

[65] Khashaba U.A., Alazwari M.A. (2025). Thermo-mechanical damage and cutting forces analysis in drilling UD-GFRP composites. Compos. Part A Appl. Sci. Manuf..

[66] Slavković V., Hanželič B., Plesec V., Milenković S., Harih G. (2024). Thermo-mechanical behavior and strain rate sensitivity of 3D-printed polylactic acid (PLA) below glass transition temperature (Tg). Polymers.

[67] Xu J., Lin T., Davim J.P. (2022). On the machining temperature and hole quality of CFRP laminates when using diamond-coated special drills. J. Compos. Sci..

[68] Shanmugam V., Babu K., Kannan G., Mensah R.A., Samantaray S.K., Das O. (2024). The thermal properties of FDM printed polymeric materials: A review. Polym. Degrad. Stab..

[69] Yarar E., Özsoy M.İ., Fidan S., Ürgün S., Bora M.Ö. (2025). Drilling machinability of glass, basalt, and hybrid epoxy composites: Thrust force, thermal load, and hole quality. Polymers.

[70] Munoa J., Beudaert X., Dombovari Z., Altintas Y., Budak E., Brecher C., Stepan G. (2016). Chatter suppression techniques in metal cutting. CIRP Ann..

[71] Altintas Y., Stépán G., Merdol D., Dombóvári Z. (2008). Chatter stability of milling in frequency and discrete time domain. CIRP J. Manuf. Sci. Technol..

[72] Quintana G., Ciurana J. (2011). Chatter in machining processes: A review. Int. J. Mach. Tools Manuf..

[73] Rahimi M.H., Huynh H.N., Altintas Y. (2021). On-line chatter detection in milling with hybrid machine learning and physics-based model. CIRP J. Manuf. Sci. Technol..

[74] Altintas Y., Stepan G., Budak E., Schmitz T., Kilic Z.M. (2020). Chatter stability of machining operations. J. Manuf. Sci. Eng..

[75] Baraheni M., Shabgard M.R., Amini S., Gholipour F. (2024). Experimental evaluation and optimization of parameters affecting delamination, geometrical tolerance and surface roughness in ultrasonic drilling of 3D-printed PLA thermoplastic. J. Thermoplast. Compos. Mater..

